# Desflurane versus sevoflurane in pediatric anesthesia with a laryngeal mask airway: A randomized controlled trial: Erratum

**DOI:** 10.1097/MD.0000000000008594

**Published:** 2017-11-03

In the article, “Desflurane versus sevoflurane in pediatric anesthesia with a laryngeal mask airway: A randomized controlled trial”,^[1]^ which appeared in Volume 96, Issue 35 of *Medicine*, Dr. Jin-Tae Kim's affiliation should have appeared as “Department of Anesthesiology and Pain Medicine, Seoul National University Hospital, Seoul National University College of Medicine, Seoul, Republic of Korea.”
